# Consensus clinical management guidelines for Friedreich ataxia

**DOI:** 10.1186/s13023-014-0184-7

**Published:** 2014-11-30

**Authors:** Louise A Corben, David Lynch, Massimo Pandolfo, Jörg B Schulz, Martin B Delatycki, On behalf of the Clinical Management Guidelines Writing Group

**Affiliations:** Bruce Lefroy Centre, Murdoch Childrens Research Institute, Parkville, 3052 Victoria Australia; Monash Health, Clayton, 3168 Victoria Australia; Department of Neurology, Children’s Hospital of Philadelphia, Pennsylvania, USA; Department of Pediatrics, Children’s Hospital of Philadelphia, Pennsylvania, USA; University of Pennsylvania, Pennsylvania, USA; Laboratory of Experimental Neurology, Université Libre de Bruxelles, Brussels, Belgium; Department of Neurology, University Hospital, Aachen, Germany; Department of Clinical Genetics, Austin Health, Heidelberg, 3084 Victoria Australia; Department of Paediatrics, Melbourne University, Parkville, 3052 Victoria Australia

**Keywords:** Friedreich ataxia, Clinical, Guidelines, Evidence, Recommendations

## Abstract

Friedreich ataxia (FRDA), a multisystem autosomal recessive condition, is the most common inherited ataxia in Caucasians, affecting approximately 1 in 29,000 individuals. The hallmark clinical features of FRDA include progressive afferent and cerebellar ataxia, dysarthria, impaired vibration sense and proprioception, absent tendon reflexes in lower limbs, pyramidal weakness, scoliosis, foot deformity and cardiomyopathy. Despite significant progress in the search for disease modifying agents, the chronic progressive nature of FRDA continues to have a profound impact on the health and well-being of people with FRDA. At present there is no proven treatment that can slow the progression or eventual outcome of this life-shortening condition. Thirty-nine expert clinicians located in Europe, Australia, Canada and USA critically appraised the published evidence related to FRDA clinical care and provided this evidence in a concise manner. Where no published data specific to FRDA existed, recommendations were based on data related to similar conditions and/or expert consensus. There were 146 recommendations developed to ensure best practice in the delivery of health services to people with FRDA. Sixty-two percent of recommendations are based on expert opinion or good practice indicating the paucity of high-level quality clinical studies in this area. Whilst the development of these guidelines provides a critical first step in the provision of appropriate clinical care for people with FRDA, it also highlights the urgency of undertaking high-quality clinical studies that will ensure the delivery of optimum clinical management and intervention for people with FRDA.

## Introduction

Friedreich ataxia (FRDA), the most common of the hereditary ataxias, is an autosomal recessive, multisystem disorder affecting approximately 1 in 29,000 individuals and has a carrier frequency of 1 in 85 in individuals of Caucasian background [1,2]. The hallmark neurological features of FRDA include progressive afferent and cerebellar ataxia, dysarthria, fixation instability, impaired vibration sense and proprioception, and pyramidal weakness. Most affected individuals have absent lower limb reflexes, but some have retained reflexes and may have spasticity. Scoliosis, diabetes, foot deformity and cardiomyopathy are common non-neurological features [3-5]. Pathology related to FRDA includes degeneration of the dorsal root ganglia and posterior columns of the spinal cord, spinocerebellar tracts, corticospinal tracts, dentate nuclei of the cerebellum and the heart [6,7]. FRDA is due to mutations in *FXN* [8]. In about 96% of mutant alleles there is homozygosity for a pathological expansion of a GAA trinucleotide repeat in intron one of *FXN* whilst in the other 4% there is compound heterozygosity for an intron 1 GAA repeat expansion in one allele and a point mutation or deletion in the other [8-10]. The GAA expansion results in a reduction of frataxin, a mitochondrial membrane protein involved in iron sulfur protein production, storage and transport [11,12]. Since the discovery of the molecular basis underlying this disorder in 1996, there has been an abundance of studies exploring the nature of the mutation, the role of frataxin, disease progression and disease modifying agents [11,13-15]. Although no specific therapies have been identified that can alter the course of this devastating disease, a number of promising compounds have been identified [11,16]. However the challenge for clinicians to provide effective, evidence-based clinical management for the multifaceted issues facing people with FRDA endures.

In 2003, “*Revalidatie Geneeskundige Richtlijn Ataxie van Friedreich*” was written by a special task force under the auspices of ‘*Vereniging Spierziekten Nederland’.* These were the first guidelines that provided an evidence base to the clinical management of people with FRDA. These guidelines were subsequently updated and adapted for international use in September 2007 (http://www.vsn.nl/hulpverleners/protocol_detail.php?protocol_id=17). In 2009, Ataxia UK launched “Management of ataxia: towards best clinical practice”, developed to provide recommendations for the management of people with inherited ataxia, including FRDA (http://www.ataxia.org.uk/pages/resources-and-publications.html). Whilst this further initiative was welcomed, it was apparent that issues specific to FRDA required disease specific guidelines. Furthermore, it was apparent that the multiple gaps in evidence surrounding service delivery may provide a platform for ongoing research.

## Method

### Assembling the Executive Committee and Specialist Working Groups

An executive committee (MBD, DL, MP, JBS and LAC) was convened to oversee the process of guideline development. Clinicians with expertise in FRDA were recruited to contribute to the guidelines through invitations from the executive. Thirty-nine individuals participated in the writing of these guidelines. This group advanced the topics and corresponding clinical questions which would be the foundation of the guidelines. Specialist working groups (SWGs) related to specific topics were established. There were two face-to-face meetings during the guideline development phase that were attended by some of the 39 authors, otherwise communication was facilitated by teleconference and email. Each member of the SWGs was asked to formally declare any potential conflict of interest however none were present that required removal of any individual from the writing groups.

### Developing topics and clinical questions

An initial topic list was developed by MBD and LAC. This list was refined by discussion with the executive and the SWGs (see Table 1 for the final topic list). The specific topics within the guidelines comprised a description of the topic, associated natural history, investigations, an evaluation of the evidence and graded recommendations. Where possible, clinical review questions were developed around the PICO framework (patients/population, intervention, comparison and outcome) [17] which formed the basis of the examination of available evidence.

**Table 1:** **List of topics included in the clinical management guidelines**

1	Overview of Friedreich Ataxia
2	The neurological components of Friedreich Ataxia
2.1	Ataxia
2.2	Weakness
2.3	Neuropathy
2.4	Spasticity and muscle spasm
2.5	Restless legs
2.6	Mobility
2.7	Dysarthria
2.8	Dysphagia
2.9	Vision
2.10	Bladder function
2.11	Bowel function
2.12	Sexual function
2.13	Audiological function
2.14	Cognition
2.15	Rehabilitation
3	The heart, cardiovascular and respiratory systems in Friedreich Ataxia
3.1	The heart
3.2	Sleep
3.3	Pain management and anesthesia
4	Scoliosis
5	Diabetes Mellitus
6	Genetic Issues
7	Friedreich Ataxia due to compound heterozygosity for a *FXN* intron 1 GAA expansion and point mutation/insertion/deletion
8	Pregnancy issues
9	Quality of life issues
9.1	Overview of quality of life in Friedreich Ataxia
9.2	Mental health issues
9.3	Provision of wheelchairs and seating systems
9.4	Independence issues
9.5	Advance care planning and end of life
9.6	Palliative care
9.7	Potential medications/compounds for use in Friedreich Ataxia

### Literature search and evaluation of the literature

A literature review was conducted for each topic. Clinical databases included in the search were PubMed, MEDLINE, CINAHL, Best Practice, Cochrane Database of Systematic Reviews, EMBASE and Scopus. Guideline SWGs evaluated the available evidence according to the templates developed by the Guidelines International Network (http://www.g-i-n.net/) for diagnostic and intervention studies.

### Grading of evidence and recommendations

A range of international methods of grading of evidence and recommendations were reviewed. These included those recommended by the American Academy of Neurology (AAN) (USA), Scottish Intercollegiate Guidelines Network (SIGN), National Institute of Health and Clinical Excellence (NICE) (UK) and the National Health and Medical Research Council (NHMRC) Australia. Given no one method was identified as clearly superior, the evidence and subsequent recommendations were graded according to the criteria developed by the NHMRC [18] (see Table 2 for levels of evidence and Table 3 for grading of recommendations). In order to indicate the strength of the body of evidence underscoring the recommendation and to ascertain if application of the evidence may result in improved health outcomes, recommendations were allocated a grading (A-D) according to the level of evidence (I to IV) available. Recommendations allocated Grade A were underpinned by a body of evidence that can be trusted to guide practice. Grade B recommendations included those for which a body of evidence can be trusted to guide practice in most situations. Grade C recommendations comprised those for which the body of evidence provides some support but care should be taken in its application, whereas an allocation of Grade D indicated the body of evidence underlying the recommendation is weak and must be applied with caution [18]. Where no clear Level I, II III or IV evidence was available but where there was sufficient consensus within the specialist working group, good practice points (GPP) were provided. A GPP is the recommended best practice based on clinical experience and the expert opinion of the SWG. SWGs were encouraged to consult widely with colleagues and peers to ensure consistency of evidence. Every effort was made to achieve consensus within the group. However for the sections on Dysarthria and Dysphagia, the recommendations were sent to the executive group for a final independent decision. On one occasion (Genetic testing of asymptomatic minors) no consensus was reached and both viewpoints are presented. Draft iterations of the guidelines were circulated to all authors involved in producing the guidelines for comment and feedback. The final draft was sent to advocacy groups representing individuals with FRDA.

**Table 2:** **Levels of evidence and grading of recommendations (National Health and Medical Research Council 1999** [19]**)**

I	Evidence obtained from a systematic review of all relevant randomized controlled trials
II	Evidence obtained from at least one properly designed randomized controlled trial
III-1	Evidence obtained from well-designed pseudo-randomized controlled trials (alterative allocation or some other method)
III-2	Evidence obtained from comparative studies with concurrent controls and allocation not randomized (cohort studies), case–control studies, or interrupted time series with a control group
III-3	Evidence obtained from comparative studies with historical control, two or more single-arm studies, or interrupted time series without a parallel control group
IV	Evidence obtained from case series, either post-test or pre-test and post-test.

**Table 3:** **National Health and Medical Research Council grading of evidence for recommendations** [18]

**Grade of recommendation**	**Description**
A	Body of evidence can be trusted to guide practice: includes one or more level I studies or several level II studies with low risk of bias directly applied to the target population and demonstrating overall consistency of results.
B	Body of evidence can be trusted to guide practice in most situations: includes one or two studies rated as II or several level III studies with low risk of bias, directly applicable to target population, and demonstrating overall consistency of results.
C	Body of evidence provides some support for recommendation(s) but care should be taken in its application: includes studies rated as III-3 with a low risk of bias or level I or II with a moderate risk of bias, some inconsistency and applicable to target population with caveats. Population studied is different from target population however clinically sensible to apply this evidence to target population.
D	Body of evidence is weak and recommendation must be applied with caution: includes level IV or level I to IV studies with high risk of bias, inconsistent evidence and not applicable to target population.
Good practice point (GPP)	Recommended best practice based on clinical experience and expert opinion.

## Results

The guidelines comprise 9 sections and 25 subsections (see Table 1). There are 146 recommendations related to 1) the neurological components of FRDA; 2) the heart, cardiovascular and respiratory system; 3) scoliosis; 4) diabetes mellitus; 5) genetic issues; 6) FRDA due to *FXN* compound heterozygosity; 7) pregnancy issues; 8) quality of life issues. There were three recommendations graded as A, six graded as B, 28 graded as C, 17 graded as D and 92 GPP. The full guidelines are available on the internet (http://www.curefa.org/physicians.html). The following provides a summation of the recommendations from each topic (Table 4).

**Table 4:** **The neurological components of Friedreich ataxia**

**1.1 Ataxia**	
**Recommendations**	**Grade**
Regular neurological examination should take place, and may guide referral to appropriate specialists in a timely fashion.	GPP
Physical therapy may be useful to help with balance, flexibility, accuracy of limb movements, and maintenance of strength.	GPP
Occupational therapy may identify risks for people with ataxia as well as help minimize difficulties in the performance of daily activities.	GPP
Routine orthopedic care is necessary to follow and treat orthopedic issues that can influence ataxia.	GPP
**1.2 Weakness**	
**Recommendations**	**Grade**
Assessment of muscle weakness is an essential part of the functional evaluation of an individual with FRDA.	GPP
Fatigue is a prevalent symptom in FRDA that may be included in quality of life assessments of individuals with FRDA.	GPP
Physical therapy and exercise training may improve strength, motor performance and reduce fatigue.	GPP
Muscle weakness may interfere with the clinical assessment of coordination and gait in individuals with FRDA.	GPP
Medications improving mitochondrial function may improve muscle strength and reduce fatigue.	GPP
**1.3 Neuropathy**	
**Recommendations**	**Grade**
Neuropathic pain may be treated with Gabapentin, Pregabalin, Lamotrigine, Amitriptyline or Duloxetine.	C [20,21]
A detailed sensory assessment and examination will establish the extent of neuropathy.	GPP
Protective foot care is important.	GPP
Preventative measures such as review of daily activities, transfers and wheelchair positioning may reduce the incidence of focal neuropathies.	GPP
**1.4 Spasticity and muscle spasm**	
**Recommendations**	**Grade**
People with FRDA may benefit from assessment for spasticity, pain and spasms (including nocturnal spasms) and incipient or established contracture. This may guide treatment.	GPP
On implementation of an anti-spasticity intervention, individuals with FRDA may benefit from reassessment as the treatment of spasticity can unmask weakness and cause deterioration in gait and standing transfers. Individuals should be warned of this phenomenon before anti-spasticity interventions are commenced.	GPP
Aggravating factors such as infection, pain, constipation, diarrhea, dehydration and pressure sores should be considered and treated in the context of acute onset or exacerbation of spasticity and/or ataxia.	GPP
Spasticity and spasms should be treated at an early stage, initially by non-pharmacological means. If these are unsuccessful, pharmacological means such as the use of Baclofen, Tizanidine, Benzodiazepines, Dantrolene sodium, Gabapentin, botulinum toxin injections, alcohol and phenol injections or intrathecal Baclofen pumps may be considered. The benefit of such compounds must be balanced against adverse effects they might produce on other symptoms of FRDA (for example ataxia). In the last resort, surgical options may be considered. The distribution of spasticity around the body may also determine which intervention is chosen.	C [22-24]
Individuals with FRDA, families and caregivers should be educated to monitor the development of spasticity and incipient contractures, and should be given an ongoing plan of exercises and passive or active stretching to be performed routinely outside the clinical setting.	C [22]
**1.5 Restless legs (RLS)**	
**Recommendations**	**Grade**
People with FRDA should be specifically asked if they have RLS symptoms.	B [25,26]
A full history of the symptoms should be taken from patients suspected of having RLS so that other confounding conditions i.e. periodic leg movements can be excluded.	B [27]
Secondary causes of RLS should be excluded, in particular, a drug history should be taken and serum ferritin measurement should be undertaken.	B [27]
Initial treatment of RLS should consider the needs of the patient, the severity of the symptoms, the relative significance of the reported effects of the treatment and the level of dysfunction attributable to RLS.	A [28]
**1.6 Mobility**	
**Recommendations**	**Grade**
Mobility, balance, core stability, trunk control, spasticity, foot position and strength should be assessed by a suitably qualified physical therapist.	GPP
The impact of spasticity of lower limbs on mobility should be evaluated when assessing gait.	GPP
Foot and ankle posture should be assessed by a suitably qualified physical therapist and treated proactively.	D [5,29]
Strategies such as an appropriate exercise program, aquatic physical therapy and stretches may be implemented to prolong ambulation and reduce the number of falls in people with FRDA.	D [30,31]
Individuals with FRDA dependent on a wheelchair for mobility may still benefit from rehabilitation to improve their mobility.	D [31]
Botulinum toxin and prescription of ankle-foot orthotics may be useful in reducing the impact of spasticity during mobility and will help maintain good foot alignment for mobility.	GPP
Gait aid provision may prolong the capacity to walk. A heavy/weighted gait-aid may be a beneficial for some individuals with FRDA.	GPP
Standing frame and tilt table may be used to maintain foot alignment to enable independent transfers.	GPP
An inpatient rehabilitation program may prolong mobility and transfer ability.	D [31]
**1.7 Dysarthria**	
**Recommendations**	**Grade**
People with FRDA should undergo a comprehensive communication evaluation by a speech and language pathologist at the time of diagnosis or symptom onset and thereafter undertake review assessments to monitor performance.	C [32]
Instruction in environmental modification may be beneficial for individuals with motor speech difficulties.	C [33]
Participation in intensive and systematic behavior therapy may be beneficial to people with FRDA with dysarthria.	C [33]
Traditional non-systematic behavioral therapy may not be helpful for mitigating the effects of progressive dysarthria.	GPP
**1.8 Dysphagia**	
**Recommendations**	**Grade**
People with FRDA should undergo a comprehensive swallowing evaluation by a speech and language pathologist at the time of diagnosis or symptom onset and thereafter to monitor performance.	D [34,35]
Instruction in environmental modification and compensatory postures may be beneficial for individuals with dysphagia.	D [36-39]
Instruction in dietary modification may be beneficial for individuals with dysphagia.	D [38,40,41]
Traditional non-systematic behavioral therapy (e.g., oral motor therapy) may not be helpful for mitigating the effects of dysphagia.	GPP
**1.9 Vision**	
**Recommendations**	**Grade**
Screening or testing as per country specific general vision screening guidelines should be applied to individuals with FRDA.	GPP
Memantine, Acetazolamide, Aminopyridine, Clonazepam, Gabapentin or Ondansetron may be of benefit in treating square wave jerks and ocular flutter.	D [42]
**1.10 Bladder dysfunction**	
**Recommendations**	**Grade**
Exclusion of a concomitant urinary tract infection and assessment of post micturition residual urine is recommended prior to commencement of treatment.	C [43,44]
Antimuscarinic medications may be considered for people with FRDA displaying overactive bladder symptoms.	GPP
Intradetrusor injections of Botulinum toxin A or suprapubic catheterization may be considered as alternative intervention.	GPP
In a patient with persistently elevated post void residual volumes in excess of 100 mL, clean intermittent self-catheterization is indicated.	GPP
**1.11 Bowel dysfunction**	
**Recommendations**	**Grade**
Consider modifying diet and lifestyle to optimize stool consistency and avoid fecal incontinence.	GPP
Titrate appropriate laxatives to optimize gut transit, stool consistency and avoid fecal impaction. Consider the use of prokinetic drugs.	GPP
Avoid fecal incontinence by treating fecal impaction if present. Facilitate prompt rectal evacuation via use of manual maneuvers and/or use of suppositories/mini enemas. Consider use of transanal irrigation and biofeedback behavioral therapy.	GPP
**1.12 Sexual function**	
**Recommendations**	**Grade**
Individuals with FRDA may benefit from discussion regarding their sexual function.	GPP
Reported sexual dysfunction should be investigated.	GPP
Symptomatic management of erectile dysfunction involves the use of phosphodiesterase-5 inhibitors but should only be prescribed in an individual with cardiac disease after consultation with the individual’s cardiologist.	GPP
**1.13 Audiological function**	
**Recommendations**	**Grade**
People with FRDA should undergo a comprehensive auditory evaluation at the time of diagnosis and thereafter annually undertake a hearing screen or sooner if warranted by a sudden change in auditory performance.	B [45]
Instruction in “listening tactics” may be beneficial for individuals with hearing difficulties.	B [46]
FM-listening devices fitted by an audiologist may improve day-to-day listening and general communication in individuals with FRDA.	B [47]
Conventional hearing aids and cochlear implants may not improve the hearing impairment related to FRDA.	GPP
**1.14 Cognition**	
**Recommendations**	**Grade**
Consideration should be given to changes in cognitive function that may impact on independence.	GPP
The impact of cognitive capacity on academic skills should be considered in academic environments.	GPP
**1.15 Rehabilitation**	
**Recommendations**	**Grade**
Intensive inpatient rehabilitation is beneficial in improving function for people with FRDA.	C [31]
People with FRDA may require a cardiologist opinion prior to undergoing aquatic physical therapy.	GPP
Rehabilitation may be provided in various home or community based settings.	GPP
Rehabilitation should be provided by allied health staff with expertise in neurological conditions.	GPP
People with FRDA may benefit from maintenance rehabilitation and regular review of function.	GPP
**2 The heart, cardiovascular and respiratory systems in Friedreich Ataxia**	
**2.1 The heart in Friedreich Ataxia**	
**Recommendations**	**Grade**
**Cardiac evaluation and non-drug therapy**	
An EKG and an echocardiogram should be performed at diagnosis and then at least annually.	GPP
A Holter and/or Loop monitor assessment should be performed if an individual with FRDA has palpitations.	GPP
Evaluation by a cardiologist should take place if an individual with FRDA has cardiac symptoms or abnormal results on cardiac testing.	GPP
Evaluation by a cardiologist should take place prior to major surgery.	GPP
Cardiac monitoring should take place during major surgery.	GPP
Major surgery should be conducted in a center with cardiac intensive care facilities.	GPP
Exercise therapy, including structured aerobic exercise and light weights, is recommended.	GPP
Heavy weight training is not advised.	GPP
**Pharmacological therapy for slowing or prevention of deterioration of left ventricular contraction in asymptomatic individuals with reduced ejection fraction**	
An angiotensin converting enzyme inhibitor (Enalapril, Ramipril, Lisinopril or Trandolapril) is first line therapy but if the angiotensin converting enzyme inhibitor is not tolerated then an angiotensin 2 receptor blocker (Candesartan, Valsartan) should be commenced instead (second line therapy).	C [48]
Beta blockers (Carvedilol, Bisoprolol or long acting Metoprolol) should be considered as an addition to an angiotensin converting enzyme inhibitor or angiotensin 2 receptor blocker, particularly if the heart rate is >75/min.	C [48]
**Pharmacological therapy for treatment of symptomatic heart failure with reduced LV ejection fraction**	
A diuretic should be prescribed for fluid overload.	C [48]
An angiotensin converting enzyme inhibitor (Enalapril, Ramipril, Lisinopril or Trandolapril) is first line therapy but if the angiotensin converting enzyme inhibitor is not tolerated then an angiotensin 2 receptor blocker (Candesartan, Valsartan) should be commenced instead (second line therapy).	C [48]
Beta blockers (Carvedilol, Bisoprolol or long acting Metoprolol) should be added (first line therapy) to the angiotensin converting enzyme inhibitor or angiotensin 2 receptor blocker, however the role of beta blockers in children is less clear.	C [48]
Spironolactone or Eplerenone should be considered for individuals with New York Heart Association (NYHA) stage 3 or 4 symptoms.	C [48]
Calcium channel blockers with negative inotropic effects (Verapamil and Diltiazem) should be avoided.	C [48]
Digoxin should be considered for control of ventricular response if atrial fibrillation is present.	C [48]
**Device therapy for subjects with symptomatic heart failure and reduced ejection fraction**	
Implantation of an automatic internal cardioverter defibrillator should be considered if left ventricular ejection fraction (LVEF) is ≤35%, the individual has NYHA functional class 2 or 3 symptoms despite receiving optimal medical therapy, and the individual has a reasonable expectation of survival with good functional status for more than 1 year.	C [49]
Cardiac resynchronization therapy should be considered in individuals with LVEF of ≤35%, sinus rhythm, a QRS duration ≥0.12 seconds and NYHA functional class 3 or 4 symptoms despite receiving optimal medical therapy.	C [49]
**Antiarrhythmic agents for prevention of recurrence of atrial arrhythmias**	
Agents which may be considered for prevention of recurrence of atrial arrhythmias are a beta blocker (Metoprolol, Bisoprolol or Carvedilol), Sotalol, Dofetilide or Amiodarone.	C [50]
Agents which should be avoided include Quinidine, Flecainide, Propafenone and Disopyramide due to their negatively inotropic and/or pro-arrhythmic effects.	C [50]
**Anticoagulation for atrial arrhythmias**	
Anticoagulation should not be commenced if the LVEF is normal and there are no other risk factors for thromboembolism	C [50]
Anticoagulation with Warfarin or one of the novel anticoagulants (Dabigatran, Rivaroxaban or Apixaban) should be considered in paroxysmal or permanent AF if one CHADS_2_ risk factor is present and will be generally indicated if more than one CHADS_2_ risk factor is present.	C [50]
Anticoagulation with warfarin or one of the novel anticoagulants (Dabigatran, Rivaroxaban or Apixaban) is strongly recommended in paroxysmal or permanent AF if there is reduced LVEF.	C [50]
**Antiarrhythmic agents for prevention of recurrence of ventricular arrhythmias**	
A beta blocker (Metoprolol, Bisoprolol or Carvedilol) should be used, but Sotalol and Amiodarone are second-line options if there is arrhythmia recurrence despite beta blocker use.	C [49]
**Cardiac transplantation**	
It is recommended that individuals with FRDA should be considered for heart transplantation if they experience severe heart failure which does not respond to maximal medical management.	GPP
**2.2 Sleep**	
**Recommendations**	**Grading**
Clinicians, caregivers and individuals with FRDA should be aware there is increased prevalence of obstructive sleep apnea (OSA) as FRDA progresses.	C [51]
Annual evaluation of presence of sleep disordered breathing may be undertaken by administering of the Epworth Sleepiness Scale and reporting of clinical symptoms.	C [51]
For individuals with FRDA there should be a lower threshold for referral to a Sleep Physician and for polysomnography.	C [51]
Nasal continuous positive airway pressure therapy should be considered in the treatment of OSA.	C [52]
**2.3 Pain management and anesthesia**	
**Recommendations**	**Grading**
Consideration should be given to appropriate management of peri-operative pain in people with FRDA.	GPP
Consideration should be given to the use of nondepolarizing muscle relaxants, in particular accurate assessment of neuromuscular block throughout anesthesia.	D [53,54]
Consideration should be given to avoiding risks associated with hyperkalemia.	GPP
There should be careful monitoring of fluid balance and cardiovascular function in people with FRDA undergoing anesthesia.	GPP
**3. Scoliosis**	
**Recommendations**	**Grading**
Individuals with FRDA with a spinal curve between 20° and 40° and/or between the ages of 10–16 years should be observed for curve progression.	GPP
Bracing may not reduce or stop the progression of curves however may be valuable in delaying surgical correction in the young child.	D [55]
People with FRDA with a scoliosis >40° may be considered appropriate for surgical correction.	D [56,57]
Consideration should be given to delaying surgical intervention in ambulant individuals with FRDA.	D [55]
All people with FRDA considered for scoliosis surgery require extensive pre-operative evaluation and planning regarding cardiac and pulmonary function.	GPP
**4. Diabetes mellitus**	
**Recommendations**	**Grading**
HbA1C may not be a good screening/diagnostic test in FRDA as it is not recommended in young individuals and in people in whom diabetes may present acutely.	GPP
Blood glucose should be measured at least once a year.	GPP
Oral glucose tolerance tests have a better sensitivity than fasting plasma glucose or HbA1c to detect early changes in glucose metabolism, and enable earlier diagnosis of diabetes.	GPP
Diabetes treatment should be initiated early.	GPP
Lifestyle changes (diet and exercise) should be implemented in all with diabetes.	GPP
Insulin therapy should be initiated if diet and exercise alone do not achieve glucose control.	GPP
**5. Genetic issues**	
**Recommendations**	**Grading**
Any individual in whom the diagnosis of FRDA is considered should undergo genetic testing for FRDA.	GPP
Referral to a clinical geneticist or genetic counselor should be considered on diagnosis of FRDA.	GPP
Requests for pre-symptomatic genetic testing are best managed on a case-by-case basis; there is no evidence to support the routine provision or refusal of pre-symptomatic genetic testing for FRDA.	GPP
The committee did not reach consensus on the issue of whether it is appropriate to conduct presymptomatic testing in a minor. Where a request for presymptomatic testing in a minor occurs, the individual/family should be referred to a team with expertise in this field for discussion about pre-symptomatic genetic testing in which the risks and benefits of pre-symptomatic genetic diagnosis are put forward. The risks and benefits from both the child’s and parents’ perspectives should be carefully reviewed during the pre-test assessment.	GPP
A multidisciplinary approach to the pre-symptomatic testing process, with the additional involvement of a psychologist or psychiatrist with expertise in pediatric and adolescent issues, and if necessary a bioethicist, should be considered.	GPP
All patients identified pre-symptomatically and their families would benefit from immediate post-test counseling and psychosocial support and referral for appropriate neurological and cardiac surveillance.	GPP
Minors who have the maturity to do so, should be involved in the decision as to whether or not they are tested.	GPP
There is no evidence to support routine use of anti-oxidant therapies, such as Idebenone in patients diagnosed pre-symptomatically.	GPP
Carrier testing should be first undertaken on the closest relative.	GPP
**6. Friedreich Ataxia due to compound heterozygosity for a** ***FXN*** **Intron 1 GAA expansion and point mutation/insertion/deletion**	
**Recommendation**	**Grading**
If a person compound heterozygous for a *FXN* GAA expansion and a point mutation/insertion/deletion has a similar phenotype to those with FRDA due to homozygosity for GAA expansions, they should be managed as per the guidelines in this document.	GPP
If spastic ataxia is the predominant phenotype, then the main management issue is that of spasticity and the guidelines for management of spasticity should be followed.	GPP
It should never be assumed that other features of typical FRDA will not be present (e.g. cardiomyopathy, diabetes) and therefore monitoring for these should take place.	GPP
**7 Pregnancy**	
**Recommendations**	**Grading**
The availability of testing for carrier status of reproductive partners should be made known to couples where one member has FRDA. If testing is requested, the carrier status of the unaffected partner should be established prior to conception in order to advise the couple of the risk of having a child with FRDA and to offer appropriate counseling.	GPP
When possible, it is advisable for women to have children earlier in their disease course.	GPP
Glucose tolerance testing should be performed between 24–28 weeks of gestation or earlier for individuals deemed to be at high risk by their practitioner.	D [58]
Women with FRDA should have close monitoring by a cardiologist during pregnancy.	GPP
Pregnant women with FRDA and deep venous thrombosis should be treated with Heparin as opposed to Warfarin.	D [59]
Vaginal delivery can be expected for most pregnancies in women with FRDA.	D [60]
Close fetal monitoring during delivery is recommended.	D [61]
If Cesarean section is medically indicated, epidural or spinal anesthesia can generally be safely used in women with FRDA.	D [62,63]
**8 Issues related to quality of life**	
**8.1 Quality of life**	
**Recommendations**	**Grading**
Adherence to the guidelines for managing FRDA may improve quality of life.	GPP
**8.2 Mental health issues**	
**Recommendations**	**Grading**
Individuals with FRDA require regular evaluation in terms of risks for developing depression and/or other mental health issues.	GPP
Individuals with FRDA may benefit from regular counseling to assist in adjusting to transitional events and possibly prevent the emergence of related depression.	GPP
Individuals with FRDA identified with depression should be treated with established interventions including counseling +/− pharmacological agents.	GPP
The risk of suicide in individuals with FRDA should be considered and managed proactively.	GPP
**8.3 Provision of wheelchairs and seating systems**	
**Recommendations**	**Grading**
Prescription of a manual or powered wheelchair or scooter should be preceded by an assessment of the home/school/work and community environment the equipment will be used in.	GPP
A comprehensive prescription of a manual or powered wheelchair or scooter should be completed by a qualified clinician familiar with the specific issues related to FRDA.	GPP
A validated assessment and evaluation tool for wheelchair and seating prescription may be used to guide the process of prescription and evaluation.	GPP
In prescribing a manual wheelchair and seating system, functional capacity should not be impeded for the sake of an anatomically correct seated posture.	GPP
Appropriate training should be provided regarding the safe use of the wheelchair or scooter in the home or community environment.	GPP
Suitability of the seating and wheelchair system should be evaluated on an annual basis in adults and bi-annually in children.	GPP
**8.4 Independence issues**	
**Recommendations**	**Grading**
Individuals with FRDA may benefit from a detailed assessment identifying barriers to independence.	GPP
Compensatory or remedial intervention may improve independence in individuals with FRDA.	GPP
**8.5 Advance care planning and end of life care**	
**Recommendations**	**Grading**
Professionals should facilitate advance care planning and documentation of advance care directives in individuals with FRDA.	GPP
Advance care directives documented for individuals with FRDA should be regularly reviewed by the individual in conjunction with their treating clinicians.	GPP
**8.6 Palliative care**	
**Recommendations**	**Grading**
Neurology, rehabilitation and palliative care services should develop closely coordinated working links to support people with FRDA from diagnosis to death, including: • proper flow of communication and information for patients and their families • a designated point of contact for each stage in the pathway.	GPP
Individuals with FRDA and a limited lifespan (for example, likely to die within 12 months) and/or distressing symptoms, and/or a need for end-of-life planning generally benefit from a referral to a palliative care team.	GPP
An individual identified as being in the process of dying from FRDA may benefit from ongoing access to palliative care services including symptom and pain control, psychological and spiritual support and specialist input if needed.	GPP
**9 Potential medications/compounds for use in Friedreich Ataxia**	
**Recommendations**	**Grading**
As there are no proven treatments that alter natural history it is not recommended that any pharmaceutical agent be routinely prescribed to individuals with FRDA.	A [64-67]
Idebenone is the most studied pharmacological agent in FRDA. Studies to date indicate the use of Idebenone in individuals with FRDA does not result in significant changes to neurological or cardiac status over an extended period of time.	A [64,65,67-74]

## Conclusion

The molecular basis of Friedreich ataxia was established in 1996 [8]. The time since this pivotal discovery has seen an explosion in the understanding of the underlying mutation and the pathogenesis of the condition, the phenotype and potential pharmacological interventions. Despite this surge of information, significant gaps remain in understanding the best clinical interventions for people with FRDA. In the absence of treatments that lessen the impact of the condition, it is crucial that appropriate clinical intervention is explored and documented. This paper has reported the methodology and outcome of developing clinical management guidelines for people with FRDA. In so doing it has highlighted the disparate nature of FRDA requiring considerable depth and breadth in terms of clinical management expertise.

The principle purpose of clinical management guidelines is to provide “systematically developed statements to assist the practitioner and the patient to make decisions about appropriate health care for specific clinical circumstances” [75]. In addition clinical management guidelines play a significant role in identifying the gaps in the evidence and providing direction for ongoing high-quality studies that will underpin future iterations of the guidelines. Sixty-two percent of recommendations are based on expert opinion or good practice. For example areas such as the management of diabetes mellitus in FRDA, the complexity of genetic issues associated with symptomatic and presymptomatic testing, sexual function and quality of life have little evidence to reliably inform recommendations. In addition, areas such as the management of heart issues, dysarthria, dysphagia and scoliosis have low quality evidence guiding intervention. Whilst the development of these guidelines provides a critical first step in the provision of appropriate clinical care for people with FRDA, it also highlights the urgency of undertaking high-quality clinical studies that will ensure the delivery of optimum intervention for people with FRDA. These guidelines will be reviewed every three years and it is hoped subsequent iterations will rely less on expert opinion and more on high quality clinical studies.

## Abbreviations

AAN: American Academy of Neurology
EKG: Electrocardiogram
FRDA: Friedreich ataxia
*FXN*: Frataxin
GAA: Guanine-Adenine-Adenine
GPP: Good practice point
LVEF: Left ventricular ejection fraction
NHMRC: National Health and Medical Research Council
NICE: National Institute of Health and Clinical Excellence
NYHA: New York Heart Association
OSA: Obstructive sleep apnea
RLS: Restless leg syndrome
SIGN: Intercollegiate Guidelines Network
SWG: Specialist Working Groups

## References

[1] Cossee M, Schmitt M, Campuzano V, Reutenauer L, Moutou C, Mandel JL, Koenig M (1997). Evolution of the Friedreich’s ataxia trinucleotide repeat expansion: founder effect and premutations. Proc Natl Acad Sci U S A.

[2] Delatycki MB, Williamson R, Forrest SM (2000). Friedreich ataxia: an overview. J Med Genet.

[3] Delatycki MB, Paris DB, Gardner RJ, Nicholson GA, Nassif N, Storey E, MacMillan JC, Collins V, Williamson R, Forrest SM (1999). Clinical and genetic study of Friedreich ataxia in an Australian population. Am J Med Genet.

[4] Dürr A, Cossee M, Agid Y, Campuzano V, Mignard C, Penet C, Mandel JL, Brice A, Koenig M (1996). Clinical and genetic abnormalities in patients with Friedreich’s ataxia. N Eng J Med.

[5] Schulz JB, Boesch S, Bürk K, Dürr A, Giunti P, Mariotti C, Pousset F, Schöls L, Vandan P, Pandolfo M (2009). Diagnosis and treatment of Friedreich ataxia: a European perspective. Nat Rev Neurol.

[6] Pandolfo M (2003). Friedreich ataxia. Semin Pediatr Neurol.

[7] Pandolfo M (2009). Friedreich Ataxia: the clinical Picture. J Neurol.

[8] Campuzano V, Montermini L, Molto MD, Pianese L, Cossee M, Cavalcanti F, Monros E, Rodius F, Duclos F, Monticelli A (1996). Friedreich’s ataxia: autosomal recessive disease caused by an intronic GAA triplet repeat expansion. Science.

[9] Cossée M, Dürr A, Schmitt M, Dahl N, Trouillas P, Allinson P, Kostrzewa M, Nivelon-Chevallier A, Gustavson KH, Kohlschutter A, Muller U, Mandel JL, Brice A, Koenig M, Cavalcanti F, Tammaro A, De Michele G, Filla A, Cocozza S, Labuda M, Montermini L, Poirier J, Pandolfo M (1999). Friedreich’s ataxia: point mutations and clinical presentation of compound heterozygotes. Ann Neurol.

[10] Evans-Galea MV, Corben LA, Hasell J, Galea CA, Fahey MC, du Sart D, Delatycki MB (2011). A novel deletion-insertion mutation identified in exon 3 of FXN in two siblings with a severe Friedreich ataxia phenotype. Neurogenetics.

[11] Santos R, Lefevre S, Sliwa S, Seguin A, Camadro J, Lesuisse E (2010). Friedreich ataxia: molecular mechanisms, redox considerations, and therapeutic opportunities. Antioxid Redox Signal.

[12] Pandolfo M, Pastore A (2009). The pathogenesis of Friedreich ataxia and the structure and function of frataxin. J Neurol.

[13] Forrest SM, Knight M, Delatycki MB, Paris D, Williamson R, King J, Yeung L, Nassif N, Nicholson GA (1998). The correlation of clinical phenotype in Friedreich ataxia with the site of point mutations in the FRDA gene. Neurogenetics.

[14] Pandolfo M (2002). The molecular basis of Friedreich ataxia. Adv Exper Med Biol.

[15] Puccio H, Koenig M (2000). Recent advances in the molecular pathogenesis of Friedreich ataxia. Hum Mol Genet.

[16] Kearney M, Orrell R, Fahey MC, Pandolfo M: **Antioxidants and other pharmacological treatments for Friedreich ataxia (Review).** *Cochrane Rev* 2012, **4**. 10.1002/14651858.CD007791.pub3 22513953

[17] Schardt C, Adams MB, Owens T, Keitz S, Fontelo P (2007). Utilization of the PICO framework to improve searching PubMed for clinical questions. BMC Med Inform Decis Mak.

[18] Hillier S, Grimmer-Somers K, Merlin T, Middleton P, Salisbury J, Tooher R, Weston A (2011). FORM: An Australian method for formulating and grading recommendations in evidence-based clinical guidelines. BMC Med Res Methodol.

[19] NHMRC (1999) **A Guide to the Development, Implementation and Evaluation of Clinical Practice Guideline.** https://www.nhmrc.gov.au/_files_nhmrc/publications/attachments/cp30.pdf.

[20] Attal N, Cruccu G, Baron R, Haanpää M, Hansson P, Jensen TS, Nurmikko T (2010). EFNS guidelines on the pharmacological treatment of neuropathic pain: 2010 revision. Eur J Neurol.

[21] Dharmshaktu P, Tayal V, Kalra BS (2012). Efficacy of antidepressants as analgesics: a review. J Clin Pharmacol.

[22] Olver J, Esquenazi A, Fung VS, Singer BJ, Ward AB (2010). Botulinum toxin assessment, intervention and aftercare for lower limb disorders of movement and muscle tone in adults: international consensus statement. Eur J Neurol.

[23] Katalinic OM, Harvey LA, Herbert RD, Moseley AM, Lannin NA, Schurr K: **Stretch for the treatment and prevention of contractures.** *Cochrane Database Syst Rev* 2010, Issue 9. Art. No.: CD007455. doi:10.1002/14651858.CD007455.pub2. 10.1002/14651858.CD007455.pub2 20824861

[24] Delatycki MB, Holian A, Corben L, Rawicki HB, Blackburn C, Hoare B, Toy M, Churchyard A (2005). Surgery for equinovarus deformity in Friedreich’s ataxia improves mobility and independence. Clinical Orthopaedics & Related Research.

[25] Synofzik M, Godau J, Lindig T, Schöls L, Berg D (2011). Restless legs and substantia nigra hypoechogenicity are common features in Friedreich’s ataxia. Cerebellum.

[26] Frauscher B, Hering S, Högl B, Gschliesser V, Ulmer H, Poewe W, Boesch SM (2011). Restless legs syndrome in Friedreich ataxia: a polysomnographic study. Mov Disord.

[27] Hening WA, Allen RP, Washburn M, Lesage SR, Earley CJ (2009). The four diagnostic criteria for restless legs syndrome are unable to exclude confounding conditions (“mimics”). Sleep Med.

[28] García-Borreguero D, Silber KR, Winkelman JW, Earley CJ, Högl B, Manconi M, Montplaisir J, Inoue Y, Allen RP (2013). The long-term treatment of restless legs syndrome/Willis–Ekbom disease: evidence based guidelines and clinical consensus best practice guidance: a report from the International Restless Legs Syndrome Study Group. Sleep Med.

[29] Blattner K (1988). Friedreich’s ataxia: a suggested physical therapy regimen. Clinical Management.

[30] Carr JH, Shepherd RB (1998). Neurological Rehabilitation Optimising Motor Performance.

[31] Milne SC, Campagna EJ, Corben LA, Delatycki MB, Teo K, Churchyard AJ, Haines TP (2012). Retrospective study of the effects of inpatient rehabilitation on improving and maintaining functional independence in people with friedreich ataxia. ArchivPhys Med Rehab.

[32] Folker J, Murdoch B, Cahill L, Delatycki M, Corben L, Vogel A (2010). Dysarthria in Friedreich’s Ataxia: a perceptual analysis. Folia Phoniatrica et Logopedica.

[33] Yorkston KM, Beukelman DR (1981). Ataxic dysarthria: treatment sequences based on intelligibility and prosodic considerations. J Speech Hear Disord.

[34] Rosenbek JC, Robbins JA, Roecker EB, Coyle JL, Wood JL (1996). A penetration-aspiration scale. Dysphagia.

[35] Scott A, Perry A, Bench J (1998). A study of interrater reliability when using videofluoroscopy as an assessment of swallowing. Dysphagia.

[36] Carnaby G, Hankey GJ, Pizzi J (2006). Behavioural intervention for dysphagia in acute stroke: a randomised controlled trial. Lancet Neurol.

[37] Welch MV, Logemann JA, Rademaker AW, Kahrilas PJ (1993). Changes in pharyngeal dimensions effected by chin tuck. Arch Phys Med Rehabil.

[38] Logemann JA, Gensler G, Robbins J, Lindblad AS, Brandt D, Hind JA, Kosek S, Dikeman K, Kazandjian M, Gramigna GD, Lundy D, McGarvey-Toler S, Miller Gardner PJ (2008). A randomized study of three interventions for aspiration of thin liquids in patients with dementia or Parkinson’s disease. J Speech Lang Hear Res.

[39] Rasley A, Logemann JA, Kahrilas PJ, Rademaker AW, Pauloski BR, Dodds WJ (1993). Prevention of barium aspiration during videofluoroscopic swallowing studies: value of change in posture. Am J Roentgenol.

[40] Germain I, Dufresne T, Gray-Donald K (2006). A novel dysphagia diet improves the nutrient intake of institutionalized elders. J Am Diet Assoc.

[41] Clavé P, de Kraa M, Arreola V, Girvent M, Farré R, Palomera E, Serra-Prat M (2006). The effect of bolus viscosity on swallowing function in neurogenic dysphagia. Aliment Pharmacol Ther.

[42] Thurtell MJ, Leigh RJ (2012). Treatment of nystagmus. Curr Treat Options Neurol.

[43] Coyne KS, Kaplan SA, Chapple CR, Sexton CC, Kopp ZS, Bush EN, Aiyer LP, EpiLUTS Team (2009). Risk factors and comorbid conditions associated with lower urinary tract symptoms: EpiLUTS. BJU Int.

[44] Fowler CJ, Panicker JN, Drake M, Harris C, Harrison SC, Kirby M, Lucas M, Macleod N, Mangnall J, North A, Porter B, Reid S, Russell N, Watkiss K, Wells M (2009). A UK consensus on the management of the bladder in multiple sclerosis. J Neurol Neurosurg Psychiatry.

[45] Rance G, Corben L, Barker E, Carew P, Chisari D, Rogers M, Dowell R, Jamaluddin S, Bryson RMD (2010). Auditory perception in individuals with Friedreich’s ataxia. Audiol Neurotol.

[46] Tye-Murray N (2009). Foundations of Aural Rehabilitation: Children, Adults, and Their Family Members.

[47] Rance G, Corben LA, Du Bourg E, King A, Delatycki MB (2010). Successful treatment of auditory perceptual disorder in individuals with Friedreich ataxia. Neuroscience.

[48] Hunt SA, Abraham WT, Chin MH, Feldman AM, Francis GS, Ganiats TG, Jessup M, Konstam MA, Mancini DM, Michl K, Oates JA, Rahko PS, Silver MA, Stevenson LW, Yancy CW (2009). 2009 focused update incorporated into the ACC/AHA, Guidelines for the Diagnosis and Management of Heart Failure in Adults: a report of the American College of Cardiology Foundation/American Heart Association Task Force on Practice Guidelines: developed in collaboration with the International Society for Heart and Lung Transplantation. Circulation.

[49] Epstein AE, DiMarco JP, Ellenbogen KA, Estes NA, Freedman RA, Gettes LS, Gillinov AM, Gregoratos G, Hammill SC, Hayes DL, Hlatky MA, Newby LK, Page RL, Schoenfeld MH, Silka MJ, Stevenson LW, Sweeney MO, Smith SC, Jacobs AK, Adams CD, Anderson JL, Buller CE, Creager MA, Ettinger SM, Faxon DP, Halperin JL, Hiratzka LF, Hunt SA, Krumholz HM, Kushner FG (2008). Guidelines for Device-Based Therapy of Cardiac Rhythm Abnormalities: a report of the American College of Cardiology/American Heart Association Task Force on Practice Guidelines (Writing Committee to Revise the ACC/AHA/NASPE 2002 Guideline Update for Implantation of Cardiac Pacemakers and Antiarrhythmia Devices): developed in collaboration with the American Association for Thoracic Surgery and Society of Thoracic Surgeons. Circulation.

[50] Fuster V, Rydén LE, Cannom DS, Crijns HJ, Curtis AB, Ellenbogen KA, Halperin JL, Kay GN, Le Huezey JY, Lowe JE, Olsson SB, Prystowsky EN, Tamargo JL, Wann LS (2011). ACCF/AHA/HRS focused updates incorporated into the ACC/AHA/ESC 2006 guidelines for the management of patients with atrial fibrillation: a report of the American College of Cardiology Foundation/American Heart Association Task Force on practice guidelines. Circulation.

[51] Corben LA, Ho M, Copland J, Tai G, Delatycki MB (2013). Increased prevalence of sleep disordered breathing in Friedreich ataxia. Neurology.

[52] Giles TL, Lasserson TJ, Smith BH, White J, Wright J, Cates CJ: **Continuous positive airways pressure for obstructive sleep apnoea in adults.** *Cochrane Database Syst Rev* 2006, Issue 3. Art. No.: CD001106. doi:10.1002/14651858.CD001106.pub3. 10.1002/14651858.CD001106.pub2 16437429

[53] Mouloudi H, Katsanoulas C, Frantzeskos G (1998). Requirements for muscle relaxation in Friedreich’s ataxia. Anaesthesia.

[54] Bell CF, Kelly JM, Jones RS (1986). Anaesthesia for Friedreich’s ataxia. Case report and review of the literature. Anaesthesia.

[55] Tsirikos AI, Smith G (2012). Scoliosis in Friedreich’s ataxia. J Bone Joint Surg.

[56] Allard P, Dansereau J, Thiry PS, Geoffroy G, Raso JV, Duhaime M (1982). Scoliosis in Friedreich’s ataxia. Can J Neurol Sci.

[57] Cady RB, Bobechko WP (1984). Incidence, natural history, and treatment of scoliosis in Friedreich’s ataxia. J Pediatr Orthop.

[58] Jovanovic L, Peterson CM (1985). Screening for gestational diabetes. Optimum timing and criteria for retesting. Diabetes.

[59] Armstrong BA, Howat PW (2002). Pregnancy in a woman with Friedreich’s ataxia complicated by pulmonary embolism. Austr N Z J Obst Gynaecol.

[60] Friedman LS, Paulsen EK, Schadt KA, Brigatti KW, Driscoll DA, Farmer JM, Lynch DR (2010). Pregnancy with Friedreich ataxia: a retrospective review of medical risks and psychosocial implications. Am J Obstet Gynecol.

[61] Paul RH, Miller DA (1995). Cesarean birth: how to reduce the rate. Am J Obstet Gynecol.

[62] MacKenzie WE (1986). Pregnancy in women with Friedreich’s ataxia. Brit Med J Clin Res Ed.

[63] Kubal K, Pasricha SK, Bhargava M (1991). Spinal anesthesia in a patient with Friedreich’s ataxia. Anesth Analges.

[64] Enns GM, Kinsman SL, Perlman SL, Spicer KM, Abdenur JE, Cohen BH, Amagata A, Barnes A, Kheifets V, Shrader WD, Thoolen M, Blankenberg F, Miller G (2012). Initial experience in the treatment of inherited mitochondrial disease with EPI-743. Mol Genet Metab.

[65] Lynch DR, Willi SM, Wilson RB, Cotticelli MG, Brigatti KW, Deutsch EC, Kucheruk O, Shrader W, Rioux P, Miller G, Hawi A, Sciascia T (2012). A0001 in friedreich ataxia: biochemical characterization and effects in a clinical trial. Mov Disord.

[66] Cooper JM, Korlipara LV, Hart PE, Bradley JL, Schapira AH (2008). Coenzyme Q10 and vitamin E deficiency in Friedreich’s ataxia: predictor of efficacy of vitamin E and coenzyme Q10 therapy. Eur J Neurol.

[67] Richardson TE, Kelly HN, Yu AE, JW S (2013). Therapeutic strategies in Friedreich’s ataxia. Brain Res.

[68] Hausse AO, Aggoun Y, Bonnet D, Sidi D, Munnich A, Rotig A, Rustin P (2002). Idebenone and reduced cardiac hypertrophy in Friedreich’s ataxia. Heart.

[69] Artuch R, Aracil A, Mas A, Colome C, Rissech M, Monros E, Pineda M (2002). Friedreich’s ataxia: idebenone treatment in early stage patients. Neuropediatrics.

[70] Di Prospero N, Baker A, Jeffries N, Fischbeck K (2007). Neurological effects of high-dose idebenone in patients with Friedreich’s ataxia: a randomised, placebo-controlled trial. Lancet Neurol.

[71] Pineda M, Arpa J, Montero R, Aracil A, Domínguez F, Galván M, Mas A, Martorell L, Sierra C, Brandi N, García-Arumí E, Rissech M, Velasco D, Costa JA, Artuch R (2008). Idebenone treatment in paediatric and adult patients with Friedreich ataxia: long-term follow-up. Eur J Paediatr Neurol.

[72] Lynch DR, Perlman SL, Meier T (2010). A phase 3, double-blind, placebo-controlled trial of Idebenone in Friedreich ataxia. Arch Neurol.

[73] Valasco-Sánchez D, Aracil A, Montero R, Mas A, Jiménez L, O’Callaghan M, Tondo M, Capdevila A, Blanch J, Artuch R, Pineda M (2011). Combined therapy with idebenone and deferiprone in patients with Friedreich’s ataxia. Cerebellum.

[74] Boesch S, Sturm B, Hering S, Scheiber-Mojdehkar B, Steinkellner H, Goldenberg H, Poewe W (2008). Neurological effects of recombinant human erythropoietin in Friedreich’s ataxia: a clinical pilot trial. Mov Disord.

[75] Field MJ, Lohr K (1990). Clinical Practice Guidelines: Directions for a New Program.

